# Spontaneous Cell Detachment and Reattachment in Cancer Cell Lines: An In Vitro Model of Metastasis and Malignancy

**DOI:** 10.3390/ijms22094929

**Published:** 2021-05-06

**Authors:** Elena Vargas-Accarino, Carlos Herrera-Montávez, Santiago Ramón y Cajal, Trond Aasen

**Affiliations:** 1 Patologia Molecular Translacional, Vall d’Hebron Institut de Recerca (VHIR), Vall d’Hebron Hospital Universitari, Vall d’Hebron Barcelona Hospital Campus, Passeig Vall d’Hebron 119-129, 08035 Barcelona, Spain; elena.vargas@vhir.org (E.V.-A.); herreramontavez@gmail.com (C.H.-M.); 2 Anatomía Patológica, Vall d’Hebron Hospital Universitari, Vall d’Hebron Barcelona Hospital Campus, Passeig Vall d’Hebron 119-129, 08035 Barcelona, Spain; 3 CIBER de Cáncer (CIBERONC), Instituto de Salud Carlos III, Avenida de Monforte de Lemos 3-5, 28029 Madrid, Spain; 4 Universitat Autònoma de Barcelona, 08193 Bellaterra, Spain

**Keywords:** metastasis models, floating cells, suspension cells, anoikis, NM23, mTOR, cell detachment, cell reattachment

## Abstract

There is an unmet need for simplified in vitro models of malignancy and metastasis that facilitate fast, affordable and scalable gene and compound analysis. “Adherent” cancer cell lines frequently release “free-floating” cells into suspension that are viable and can reattach. This, in a simplistic way, mimics the metastatic process. We compared the gene expression profiles of naturally co-existing populations of floating and adherent cells in SW620 (colon), C33a (cervix) and HeLa (cervix) cancer cells. We found that 1227, 1367 and 1333 genes were at least 2-fold differentially expressed in the respective cell lines, of which 122 were shared among the three cell lines. As proof of principle, we focused on the anti-metastatic gene NM23-H1, which was downregulated both at the RNA and protein level in the floating cell populations of all three cell lines. Knockdown of NM23-H1 significantly increased the number of floating (and viable) cells, whereas overexpression of NM23-H1 significantly reduced the proportion of floating cells. Other potential regulators of these cellular states were identified through pathway analysis, including hypoxia, mTOR (mechanistic target of rapamycin), cell adhesion and cell polarity signal transduction pathways. Hypoxia, a condition linked to malignancy and metastasis, reduced NM23-H1 expression and significantly increased the number of free-floating cells. Inhibition of mTOR or Rho-associated protein kinase (ROCK) significantly increased cell death specifically in the floating and not the adherent cell population. In conclusion, our study suggests that dynamic subpopulations of free-floating and adherent cells is a useful model to screen and identify genes, drugs and pathways that regulate the process of cancer metastasis, such as cell detachment and anoikis.

## 1. Introduction

Metastasis is a process by which cancer cells leave their primary site and spread to other tissues and organs to form new tumors [1]. Ninety percent of deaths from solid tumors are due to metastasis. Therefore, understanding and targeting this process is critical. Key features during epithelial cancer cell metastasis are detachment, adaptation and prevention of detachment-induced cell death (anoikis) in the absence of extracellular matrix (ECM), and subsequent reattachment [1]. This is a highly complex process regulated by numerous genes including adhesion molecules, receptor tyrosine kinases, and pathways such as the mechanistic target of rapamycin (mTOR) and RhoA signalling [1].

Animal models have been essential in order to understand the mechanisms of metastasis. However, these models have a variety of limitations including a long latency for the development of metastasis, very high costs and ethical implications. This often makes their use impractical, particularly for preliminary screens of novel candidate genes or treatment compounds.

Several advanced in vitro cancer metastasis models have also been established in recent years [2]. Typically they involve specific culture conditions, including serum-free conditions and 3D matrix systems that allow for the culture of tumor spheres [3,4] and organoids [5]. Some studies have used these approaches to specifically compare detached cells to that of the standard adherent cell population. For example, glioblastoma cells have the capacity to form neurospheres, and these show distinctive features from adherent counterparts [6]. Notably, in a recent study of ovarian cancer dissemination, a multi-omic approach was used to identify and screen genes associated with anoikis resistance by comparing adherent cells and cells forced to survive in suspension in polyHEMA-treated culture dishes [7].

Some studies have also investigated the naturally occurring floating cell population observed under standard culture conditions. Major changes in gene and protein expression patterns are likely to occur in adherent cells that detach and float in suspension as single cells or small clusters. Indeed, several studies indicate that cells grown as spheres in suspension have certain unique properties such as resistance to anoikis-mediated cell death, changes in the cell cycle, regulation of surface and adhesion molecules, cancer stem cell characteristics and the process of epithelial to mesenchymal transition [8]. For example, stimulation of the UP-LN1 carcinoma cell line with IFN-y promoted the appearance of cancer stem cells (CSCs) specifically in the floating cell population but not the adherent population, concomitant with induction of CXCR4 and enhanced migratory and invasive potential [9]. When culturing the gastric cell line SGC-7901 in serum-free medium, a subpopulation of cells formed spheres in suspension that were more tumorigenic and showed upregulation of several genes related to stemness [10]. Increased CSC marker expression was also observed in the spheroid cell population compared to adherent cells derived from ovarian serous carcinomas [11]. Primary gallbladder carcinomas grown under serum-free conditions also produce floating spheres, with increased expression of the CSC marker CD133(+), higher tumorigenic potential, and increased resistance to chemotherapeutic reagents [12]. Indeed, there are many links between stem cell properties and resistance to chemotherapy and radiotherapy. In one study, endometrial cancer cells made resistant to cisplatin became smaller and grew in a floating state, concomitant with increased expression of several drug-resistance genes. Curiously, although these cells were also more resistant to mitomycin and adriamycin, they were more sensitive to etoposide and 5-fluorouracil [13]. It is unclear why nearly all small cell lung cancer (SCLC) cell lines grow in suspension [7]. One SCLC cell line, NCI-H446, has been shown to grow with coexisting floating and adherent subpopulations, where the floating cells express much higher levels of neuron-specific enolase as well as the neuronal cell adhesion molecule NCAM [14]. The SCLC cell lines NCI-H69 and NCI-N592 also grow in suspension, but surprisingly the adriamycin-resistant subclones develop an adherent phenotype [15]. Using the non-small cell lung cancer (NSCLC) lines, which unlike SCLC typically grow as adherent cells, Gomez-Casal et al. [16] showed that A549 and H460 cells that survived ionizing radiation started to grow in spheres and expressed CSC markers, concomitant with increased levels of epithelial–mesenchymal transition (EMT) markers. In stark contrast, however, EMT was associated with the adherent subpopulation of the SCLC cell lines [17]. Other changes in gene expression during the transformation from adherent to a floating phenotype have been observed in sublines established from the rat hepatoma cell line AH7974, in which floating cells decreased the expression of extracellular matrix proteins, cell surface integrins and heparan sulphate proteoglycans [18]. Thus, the interrelationship between adherent and suspension cells implicate key cancer features related to metastasis, linking EMT, CSCs and therapeutic resistance. Dissecting this complexity may reveal additional pathways and targets to prevent metastasis.

Few genes have been identified to date that drive the process of adherence towards suspension. One study showed that the free-floating organoids from the breast cancer cell line PMC42 became adherent upon addition of epidermal growth factor (EGF), concomitant with increased expression of the cell-adhesive proteins laminin and fibronectin [19]. Identifying genes differentially expressed or driving this process may also reveal surprising new therapeutic targets. In this respect, Jensen et al. [20] showed that floating thyroid cancer cells were more resistant to serum deprivation. The tumor spheres displayed constitutively activated AKT and had increased levels of the gap junction protein connexin 43. Silencing connexin 43 expression, or inhibiting gap junctions chemically, resulted in a loss of pAKT and induction of apoptosis in the spheres [20]. The induction of anoikis (matrix deprivation-induced apoptosis) is one putative therapeutic target for treating metastatic tumors [21].

When culturing cancer cell lines on adherent tissue culture plates, the population of cells seen floating in suspension is by many researchers—especially when occurring as single cells and not clusters—considered as dead (or dying) cells and debris. Here, we show that in many cases, the majority (up to 99%) of these cells are alive, can proliferate and rapidly settle down again as adherent cells when transferred to a new tissue culture plate. Although most studies have focused on tumor spheres, we have observed that single floating cells, coexisting in typically adherent cell lines, also have unique properties. Our in vitro model of cells co-existing as adherent and spontaneously detached floating cells in many ways mimic, in a very simplistic way, some characteristics of the process of metastasis from the primary tumor to a secondary organ, including detachment, survival in suspension and reattachment. We postulated that naturally co-occurring populations of free-floating and adherent cells can be used as simplistic models to screen for genes, drugs and pathways that regulate cancer metastasis. To our knowledge, large-scale genome-wide differential transcriptomic analysis comparing adherent and floating subpopulations of the same cell line co-cultured under identical conditions has not been reported. To get a better understanding of the underlying genes and pathways associated with the transition from an adherent to a floating cell phenotype, we performed RNA sequencing in three different cancer cell lines isolated from solid epithelial tumors: HeLa (cervical carcinoma cell line), SW620 (metastatic colon carcinoma cell line isolated from a lymph node) and C33a (cervical carcinoma cell line with the potential to spontaneously metastasize). As proof of principle, we identified a group of genes and signaling pathways, which we verified to be implicated in the process of either detachment, anoikis or reattachment. We suggest this model allows for a cost-effective screening method for the identification of novel anti-metastatic compounds that either inhibit cell detachment or prevent survival in suspension.

## 2. Results

### 2.1. Presence of Floating Cells in “Adherent” Cell Lines

**Figure 1:**
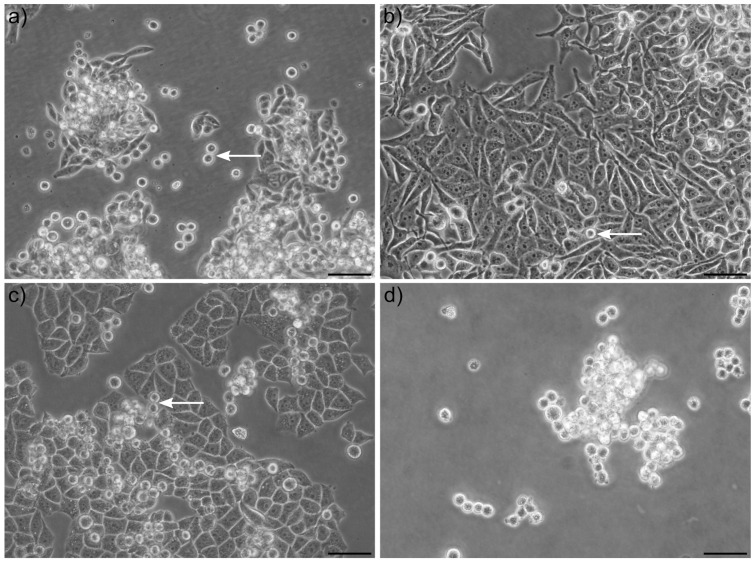
Morphology of adherent and floating tumor cells. Examples of (**a**) SW620 colon, (**b**) HeLa cervical, and (**c**) C33a cervical tumor cell lines grown as adherent cell lines with the presence of some round and detached cells (arrows) which upon transfer to a new tissue culture plate will adhere within hours and establish adherent clusters again. (**d**) Example of C33a cells grown in low-attachment treated plastic to prevent adherence, causing increased cell–cell attachment and cluster formation of the floating cell population. Scale Bars: 50 µm.

Cultured adherent cell lines sometimes release single cells into suspension (Figure 1), which by many is considered a dead or dying cell population. The amount of floating cells naturally varies highly depending on the cell line and cell confluence. We observed that the colon cancer cell line SW620 and the cervical cell line C33a produce many floating cells (Figure 1a,b) even at low density (such as the typical at 25% seeding density). To a certain extent, confluence and proliferation contribute to the dynamics of detachment, and some cells, such as HeLa cells, only release floating cells at relatively high densities (see below). Surprisingly, similar to the adherent population, the majority of floating cells are alive (98% of adherent and 94% of floating SW620 cells, and 97% of both adherent and floating C33a cells, as determined by trypan blue dye exclusion assay (Supplementary Figure S1a)). Notably, collecting the floating cell populations and placing them in a new tissue culture dish caused nearly all cells to rapidly settle down again as adherent cells, suggesting this is a highly regulated and dynamic process. However, a significant amount of floating cells appears to always be present even though there is ample space (e.g., 50%) for cells to settle down.

Low attachment plates that prevent reattachment allowed us to confirm and quantify that C33a cells also proliferated in suspension, with an estimated population doubling time of around 32 h compared to 19.4 h under adherent conditions (Supplementary Figure S1b). Notably, however, when growing cells in low-attachment dishes, fewer single cells and more adhesive sphere-like clusters of cells were observed. Critically, for this study, we did not use any specific matrix or spheroid-inducing culture system. In order to better understand the mechanics of how adherent cell culture lines produce a subset of floating cells, we analysed time-lapse images of C33a and SW620 cells for up to 48 h. This allowed us to observe that the majority, if not all of the floating cells, were produced during or shortly after cell division while cells are still rounded up. A variety of co-existing scenarios occurred: both offspring of a dividing cell would settle down as adherent, only one of the two daughter cells became adherent or both daughter cells detached as floating cells. We also observed the sudden spontaneous attachment of floating cells. Examples of time-lapse movies and their analysis can be seen in Supplementary Video 1 (includes floating C33a cells suddenly settling down and then dividing, see arrow) and Supplementary Video 2 (includes SW620 cells that divide and then detaches into the medium, see arrow). These examples are highlighted in the associated Supplementary Figure S2.

In contrast to SW620 and C33a cells, HeLa cells displayed a much higher tendency to adhere to the dish, and mainly released cells into suspension under high confluence (Figure 1c). Nevertheless, the floating cell population could be placed in a new dish and would settle down and grow as adherent cells again. We repeated this process multiple times: only collecting the floating cells and placing them into a new dish, and subsequently collecting the new floating cells after a minimum of one week in culture (changing media several times). Upon each passage, the adherent cells more readily shed viable cells into suspension. Collections of floating cells over 11 continuous passages led to the establishment of a HeLa cell line with a notably higher propensity to form a floating cell population (including at lower cell densities), and we termed this “sub-line” HeLaF11. This suggests there is some plasticity with regards to this process.

The dynamic process of detachment and reattachment observed in all three cell lines prompted us to investigate further how this process was regulated at the molecular level, initially through an RNA sequencing (RNA-seq) approach.

### 2.2. Differential Gene Expression Comparing Floating vs. Adherent Cell Populations

**Figure 2:**
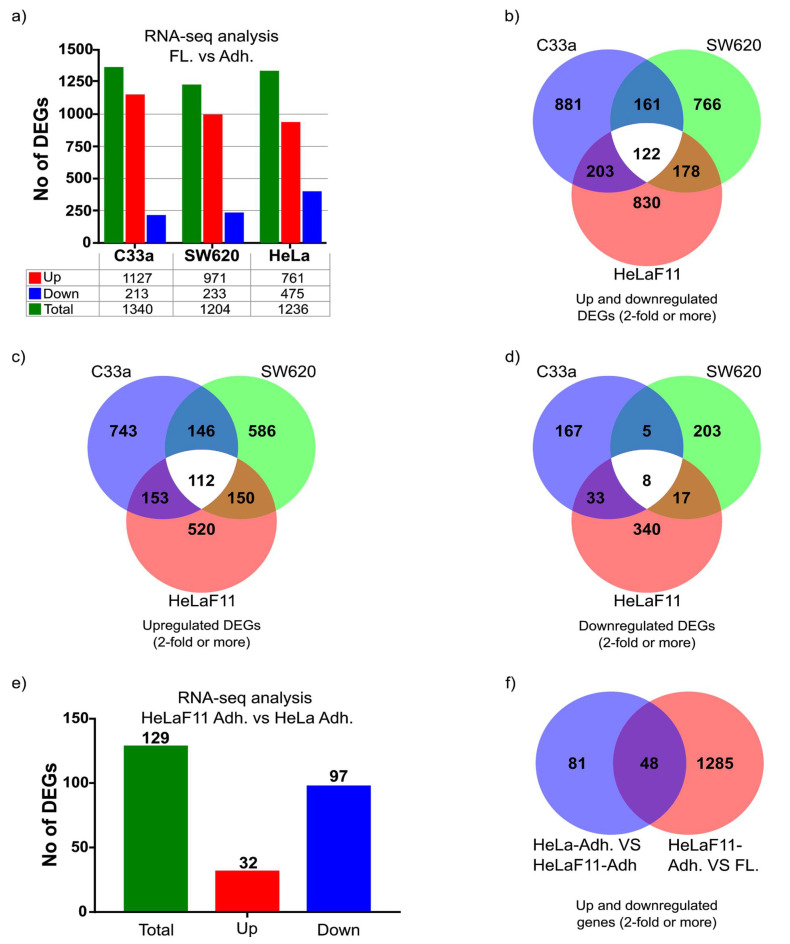
Differential gene expression between adherent and floating cells. (**a**) A table summarizing the number of differentially expressed genes (DEGs) with a minimum 2-fold difference between floating (FL.) and adherent (Adh.) cell populations. (**b**) Venn diagram displays the shared genes between cell types where there is at least 2-fold differential expression between floating and adherent cell populations. (**c**) Venn diagram displays the shared genes between cell types where there is at least 2-fold upregulated expression in the floating populations compared to the adherent cell populations. (**d**) Venn diagram displays the shared genes between cell types where there is at least 2-fold downregulated expression in the floating populations compared to the adherent cell populations. (**e**) A table summarizing the number of DEGs with a minimum 2-fold difference between adherent cells from the adapted HeLaF11 cells (two days post-adherence) and adherent cells from the parental HeLa cells. (**f**) Venn diagram displays the shared genes between where there are at least 2-fold DEGs between both adherent (Adh.) HeLa and HeLAF11 cells and between HeLaF11 adherent and HeLaF11 floating (F.) cell populations.

To minimize confounding factors, we extracted total RNA from floating and adherent cells that had been isolated from the same tissue culture dish. A summary including total reads of all genes from all samples can be found in Supplementary Table S1. A key summary of 2-fold or more differentially expressed genes (DEGs) of the RNA-seq analysis is shown in Figure 2. In the three cell lines, 1227 (SW620), 1367 (C33a) and 1333 (HeLaF11) genes showed a 2-fold or more differential expression comparing floating with adherent cells (Figure 2a–d). These genes and their level of expression are listed for each cell line in Supplementary Tables S2–S4. HeLa cells showed a higher percentage of downregulated genes. Nevertheless, comparing the three cell lines, 122 genes were found to be differentially expressed in all three cell lines (Figure 2a). These shared genes are listed in Supplementary Table S5. The high correlation of the DEGs between cell lines was encouraging (with approximately 10% of DEGs shared), suggesting a relatively common mechanism of adaptation to suspension exists in different cell types.

#### Differential Expression of Genes Comparing Parental Adherent HeLa Cells to Adapted Adherent HeLaF11 Cells

We also extracted RNA from the adherent population of the original HeLa cells and compared this to the expression profile of adherent HeLaF11 cells. RNAseq analysis revealed 129 differentially expressed genes DEGs, the majority as downregulated (Figure 2e and Supplementary Table S6). Out of these 129 DEGs, as many as 48 genes were also identified to be differentially expressed in the adherent versus the floating population of HeLaF11 cells (Figure 2f). This suggests that many genes involved in the process of adapting to a floating stage can be deregulated over time and occur in the adherent state. One could speculate that such a change in gene signature may be predictive of the propensity to metastasis.

We then decided to pick specific genes and pathways of interest, to see if we could modulate the dynamics of adherent versus floating states.

### 2.3. Proof of Principle Validation: Nm23-H1

**Figure 3:**
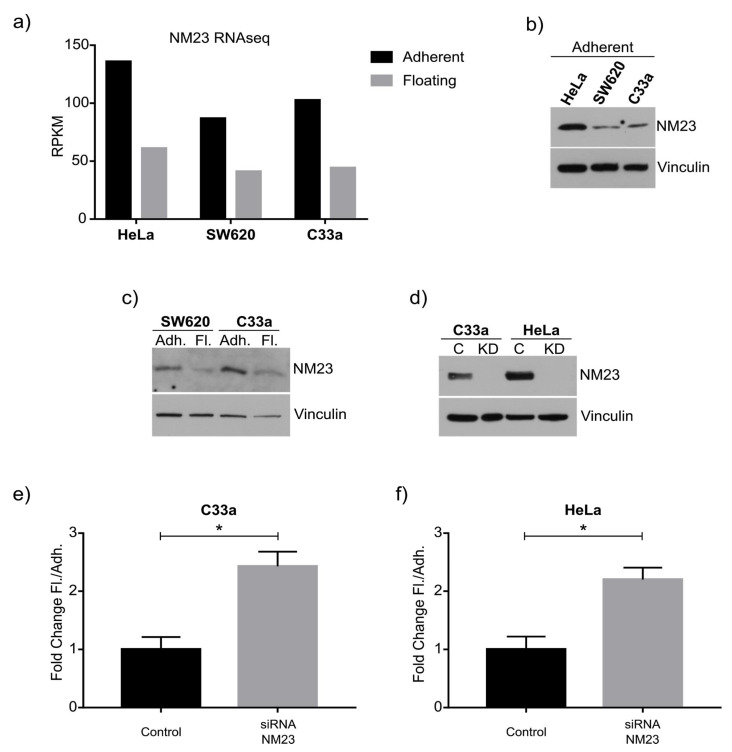
Correlation between NM23-H1 and adherent and floating cell populations. (**a**) Table display of normalized gene expression levels RPKM (Reads Per Kilobase of transcript per Million mapped reads) of NM23-H1 as identified by RNA-sequencing, showing highest expression in adherent HeLa cells and consistent downregulation in the floating cell populations. (**b**) Western blot of NM23-H1 of adherent cells showing the highest expression in HeLa cells. Vinculin was used as a loading control to verify our protein quantification and loading. (**c**) Western blot of NM23-H1 comparing the floating and adherent cell population of SW620 and C33a cells. (**d**) Western blot after control (C) or siRNA-mediated knockdown (KD) of NM23-H1 in adherent C33a and HeLa cells. (**e**–**f**) Quantification of fold changes in the number of floating cells compared to adherent cells, showing a significant increase in C33a cells (**e**) and HeLa cells (**f**) upon NM23-H1 knockdown. Graphs indicate standard error of the mean (SEM). * indicates statistical significance with a *p*-value below 0.05 based on an unpaired two-sided Student’s *t*-test of *n* = 3 independent experiments.

We decided to focus on non-metastatic gene 23 (NM-23, also known as NME1), specifically the best-known isoform Nm23-H1, a gene implicated in multiple features of metastasis including anoikis and cell attachment [22,23,24]. We chose this gene since it is known to regulate malignant features such as anoikis, and indeed it was the first anti-metastatic gene identified, and critically because our gene expression analysis showed it was one of the few genes downregulated in the floating cell population of all three cell lines (Figure 3a). The amount of NM23-H1 was confirmed at the protein level (Figure 3b) to correlate to the RNAseq analysis. The more adherent HeLa cell line expressed higher levels compared to SW620 and C33a cells. Moreover, as predicted from the RNAseq data we observed a small but noticeable downregulation of NM23-H1 in the floating cell populations (Figure 3c), with NM23-H1 becoming very scarce in the floating cell population.

We tested the hypothesis that NM23-H1 not only correlated but played a causative role in the formation of floating cells. We initially performed siRNA-mediated knockdown of NM23-H1 in HeLa cells and C33a cells, which efficiently inhibited protein expression (Figure 3d). Downregulation of NM23-H1 caused a significant increase in the ratio of floating cells in both C33a (Figure 3e) and HeLa cells (Figure 3f).

**Figure 4:**
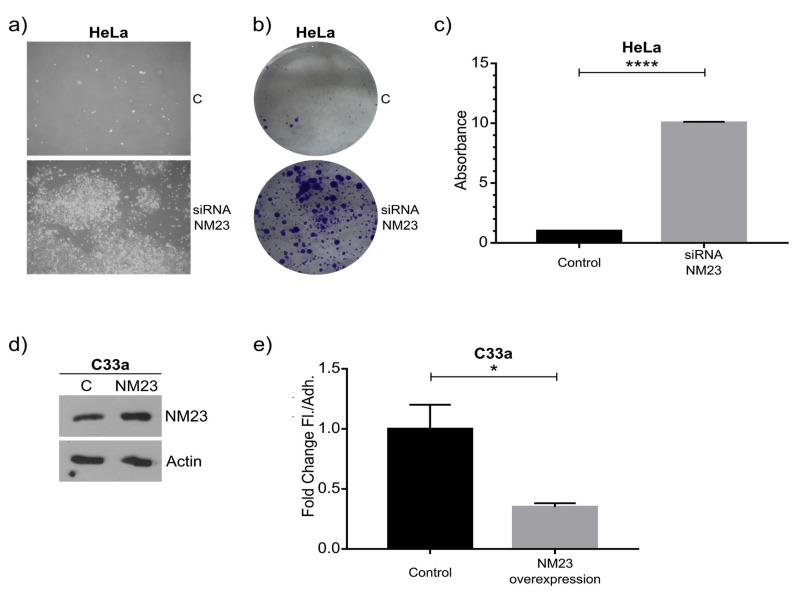
Functional consequences upon modulation of NM23-H1 expression. (**a**–**c**) 72 h after transient siRNA-mediated knockdown of NM23-H1 in HeLa cells the floating cell population was collected and transferred to a new dish. The number of cells that have survived after 2 additional days is significantly higher upon NM23-H1 knockdown (includes floating and adherent cells). The number of adherent colonies established (7 days post-transfection, with NM23-H1 expression expected to largely recover from transient siRNA knockdown at this late stage) is shown by crystal violet staining (**b**) and was statistically significant upon quantification (**c**). (**d**) Western blot of NM23-H1 of C33a cells upon control or NM23-H1 overexpression (calculated as 1.7-fold increased). (**e**) Quantification of fold change changes comparing the number of floating cells compared to adherent cells showing a significant decrease of floating cells upon NM23-H1 overexpression. Graphs indicate standard error of the mean (SEM). **** indicates statistical significance with a *p*-value below 0.0001 based on an unpaired two-sided Student’s *t*-test of *n* = 2 independent experiments. * indicates statistical significance with a *p*-value below 0.05 based on an unpaired two-sided Student’s *t*-test of *n* = 3 independent experiments.

The floating cells remained viable during the transient knockdown of NM23-H1 and after 2 days the floating HeLa cells were transferred to a new dish (5 days after transfection). The amount of floating and re-attached cells was significantly higher in NM23-siRNA cells compared to control-siRNA cells (Figure 4a). We next investigated if this transient knockdown would affect subsequent steps that occur during metastasis: reattachment and growth. The amount of cells that could re-adhere and grow as new colonies within a week is shown in Figure 4b and is quantified in Figure 4c, showing that a significantly increased number of floating cells can reattach after transient inhibition of NM23-H1 expression. This was expected since we found most cells to be alive, and, critically, since our knockdown of NM23-H1 was achieved using transient siRNA technology that gradually allows re-expression of NM23-H1. This correlates with the idea that NM23-H1 may be implicated specifically as a repressor of the transient metastatic process. From a therapeutic standpoint we, therefore, decided to overexpress NM23-H1 in C33a cells as these cells express less NM23-H1 and produce a high ratio of floating cells. As shown in Figure 4d,e, moderate physiologically relevant overexpression of NM23-H1 (approximately 1.7-fold) significantly reduced the number of floating cells. This is consistent with our hypothesis that NM23-H1 is causative in the regulation of floating versus adherent cellular states, which correlates with other reports including evidence that NM23-H1 regulates cell–cell adhesion and adherens junction proteins such as E-cadherin [25]. Indeed, increased cell-to-substrate adhesion has been shown in other cancer cells upon overexpression of NM23-H1 [26]. In the future, it would also be of interest to assess whether there is a heterogeneous expression of NM23-H1 in adherent cells that influences which cells will detach. In conclusion, NM23-H1, as a proof of principle, validated our hypothesis that specific genes regulating the metastatic process can be identified and analyzed in our model.

### 2.4. Pathways and Gene Analysis

Apart from looking at select genes of interest, we also performed pathway analysis using publicly available tools in order to identify pathways or key genes that are dysregulated in our model and may be linked to metastasis. We compared C33a and SW620 cells, as these cells readily produce floating cells and have 267 DEGs, a decent number of genes for online pathway analysis (Figure 2b). Although a systematic pathway analysis is required in the future, our examples provide proof of principle that the model system can be tested.

#### 2.4.1. Hypoxia

**Figure 5:**
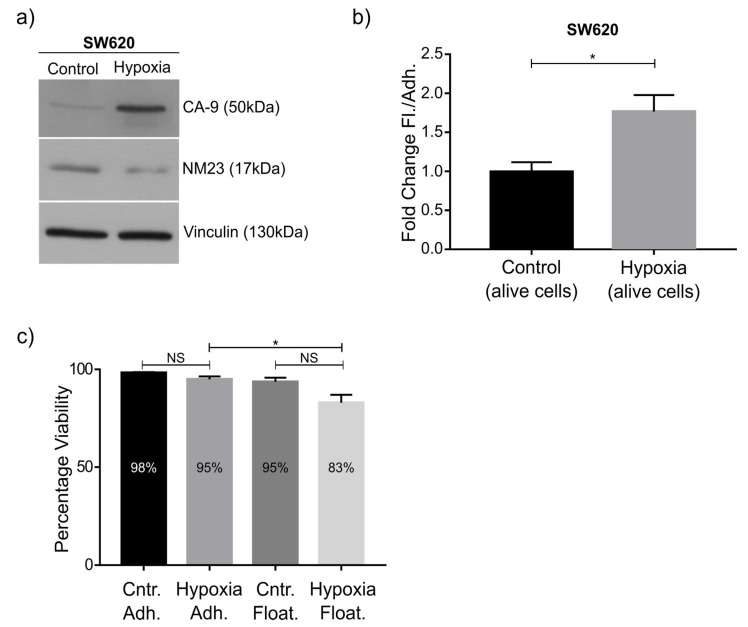
Treatment of SW620 cells to hypoxia. (**a**) Western blot showing upregulation of the hypoxia marker carbonic anhydrase 9 (CA-9) after growing SW620 cells for 48 h under hypoxic conditions (0.5% oxygen). Downregulation of NM23-H1 was observed (calculated as 2.1-fold). (**b**) Quantification of fold changes in the number of floating cells compared to adherent SW620 cells, showing a significant increase in the ratio of floating cells upon hypoxia. (**c**) No statistically significant change in the number of dead cells was observed for either adherent or floating cell populations under hypoxic conditions. Graphs indicate standard error of the mean (SEM). * indicates statistical significance with a *p*-value below 0.05 based on an unpaired two-sided Student’s *t*-test (**b**) and one-way ANOVA with Tukey’s multiple comparisons test (**c**) of *n* = 3 independent experiments.

Hypoxia is associated with a worse prognosis and is a well-known contributor to cancer metastasis [27]. Analysis of our RNAseq data using online (http://genepattern.broadinstitute.org, accessed on 7 May 2019) gene pattern analysis [28] identified several hallmarks of cancer pathways, most significantly hypoxia with 22/200 genes in overlap, *p*-value 2.71 × 10^−21^ (Supplementary Figure S3). To validate this observation, SW620 cells were subjected to hypoxic conditions (0.5% oxygen) for 48 h. The hypoxia marker CA-9 was upregulated as expected (Figure 5a). We measured the viability and the number of floating and adherent cells and observed a highly significant increased ratio of floating (and alive) cells (Figure 5b). Indeed, under hypoxic conditions, there was only a moderate decrease in cell viability, specifically in the hypoxic floating population, which was statistically different to the hypoxic adherent population (Figure 5c). Curiously, NM23-H1 was (approximately 2.1-fold) downregulated in hypoxia, consistent with our RNAseq data and our observation that NM23-H1 knockdown and overexpression affect the ratio between floating and adherent cells (Figure 3 and Figure 4). This observation is supported by recent studies that show hypoxia reduces NM23-H1 expression in multiple cancer cell lines and that this can facilitate EMT (reduced adhesiveness) and metastasis [29,30]. Indeed, EMT was also identified by pathway analysis as significantly altered (Supplementary Figure S3).

#### 2.4.2. Floating Cells Are Specifically Sensitive to Cell Death upon mTOR and ROCK Inhibition

**Figure 6:**
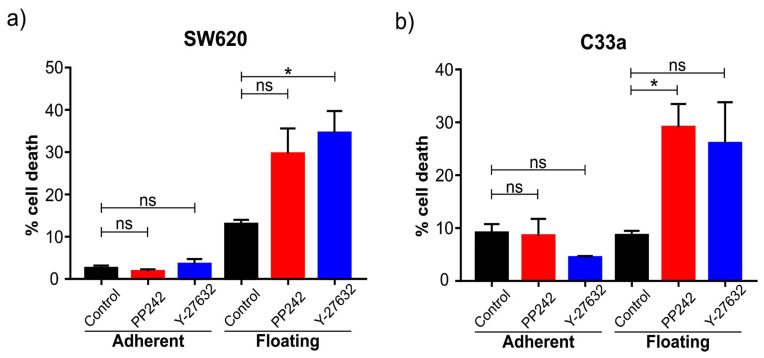
Cell death of adherent and floating cells in response to pathway inhibitors. SW620 cells (**a**) and C33a cells (**b**) were treated for 6 h with either the mTOR inhibitor PP242 or the ROCK inhibitor Y-27632. Compared to control cells (DMSO vehicle-treated), a significant increase in cell death was observed in the floating cell populations but not the adherent cell populations. Graphs indicate standard error of the mean (SEM). The star (*) indicates statistical significance with a *p*-value below 0.05 based upon ordinary one-way ANOVA with Tukey’s multiple comparisons test. *n* = 3 independent experiments.

Our pathway analysis identified that both the RhoA-ROCK pathway and the mTOR pathway were significantly modulated when comparing floating and adherent cells (Supplementary Figures S4 and S5). This was reassuring considering both pathways have been linked to cancer progression and specifically to detachment-induced apoptosis (anoikis) [1]. We, therefore, treated coexisting populations of adherent and floating cells with either the ROCK inhibitor Y-27632 or the mTOR inhibitor PP242 and measured the amount of cell death as early as 6 h post-treatment. In both SW620 and C33a cells, a significantly increased amount of cell death was observed in the floating cell population upon ROCK or mTOR inhibition (Figure 6). Notably, the viability of the adherent cell population was not significantly affected by our short treatment setting. This shows the potential of our model to identify potent drug targets, particularly for cancer cells that are in suspension during the process of metastasis, for example, those cells circulating in the bloodstream, or indeed for cancers naturally growing in suspension (e.g., leukemia).

Considering that mTOR inhibition is currently being explored in numerous clinical trials for cancer therapy, we decided to verify this finding by a second approach. Flow cytometry analysis of PP242-treated C33a cells confirmed the induction of cell death specifically in the floating cell population (Supplementary Figure S6) but also provided insight into the type of cell death. Upon mTOR inhibition, 64% of floating cells were alive compared to 93% of adherent cells. Only 17.5% of the floating cells were positive for the apoptosis marker Annexin-V, with an additional 18.2% being positive only for the cell death marker propidium iodide. This suggests that some (but not all) cells may also undergo caspase-independent cell death, a feature previously described for cells undergoing anoikis due to ECM detachment [31]. This, and the fact that a significant amount of cells seemed to undergo necrosis (18.2%), may indicate that mTOR inhibition induces cell death in floating cells via multiple pathways. However, it should be noted that this may be a highly context- and cell type-dependent feature, in part due to the complex anoikis pathways [32]. Indeed, mTOR inhibition has been suggested to protect against anoikis [33].

## 3. Discussion

Metastasis is the major killer in patients with cancer. There is a lack of simple models of malignancy and metastasis that facilitate fast, affordable and scalable in vitro gene and compound analysis. Detachment, survival in suspension, and reattachment in vitro are properties that are, in a simplistic view, mimicking the process of metastasis from primary tumors to a secondary site. To our knowledge we have for the first time performed RNA sequencing in order to compare the gene-expression profile of adherent versus floating cells that co-exist in the same tissue culture dish. We analyzed these sequencing data in order to select some genes and pathways for further validation including by Western blot, knockdown, drug modulation and by using modified culture conditions. The analysis and proof-of-concept experiments have solidified our view that this simple model is useful for cancer research to identify and screen genes, drugs and pathways that regulate either cell detachment, survival in suspension, or reattachment, which are key events in cancer metastasis with potential for anti-metastatic drug target development.

Our analysis suggests there are significant differences between cell lines in their tendency to release floating cells. Moreover, a significant amount of C33a and SW620 floating cells appears to be present despite ample space (e.g., 50%) for cells to settle down, which suggests there is an interplay between adherent and floating cells that can regulate this balance. Further analysis of DEGs from our study, particularly those of secreted factors, may provide clues towards these regulatory mechanisms.

In general, we observed a higher presence of floating cells in aggressive tumor cells (and less or none in primary cells and non-malignant cell lines). C33a has been shown to metastasize [34] and SW620 is a well-known metastatic cell line isolated from the lymph node. Moreover, we have observed that non-malignant primary fibroblasts and immortalized HaCaT keratinocytes release very few cells with the capacity to settle down and grow again, whereas a more malignant cell line such as the well-known breast cancer cell line MDA-MB-231 seems to behave more as our SW620 and C33a cell models. On the other hand, malignant MCF-7 and BT-20 breast cancer cells seem much less likely to release cells that can settle down and form new colonies. We have also observed that floating cells can form more clonogenic colonies than the equivalent amount of adherent cells, one of many features that should be explored in the future to clarify if there is a direct correlation between the ratio of adherent and floating cells to malignancy. Comparing the gene-expression profile and malignant potential of HeLa cells and HeLaF11, cells may provide interesting clues towards this unexplored hypothesis. Such comparison can also provide useful insight into which genes regulate cell detachment. As an example, from the RNA-seq analysis, we observed significantly reduced gene expression of laminin subunit beta 3 (HeLaF11 compared to HeLa cells), a well-known regulator of cell adhesion (Supplementary Table S6).

As a proof of principle of our model, we focused on NM23-H1, as this anti-metastatic gene has been robustly linked to metastasis, and was downregulated in the floating cell population of all three cell lines. Western blot confirmed the RNAseq data and knockdown and overexpression confirmed its role in regulating the ratio between adherent and floating cells. The mechanism behind this remains to be studied in detail, although previous studies have shown NM23-H1 to regulate adherens junctions [25] as well as cell–substrate adhesion [26]. Consistent with previous reports [29], NM23-H1 was also downregulated upon hypoxia, a condition that induced the ratio of floating cells. In the future it would be of interest to see whether the amount of extracellular NM23-H1 also regulates the ratio between floating and adherent cells, seeing that most floating cells seem to rapidly adhere upon transfer to a new tissue culture plate. In addition to NM23-H1, other gene candidates are being investigated. For example, in relation to EMT, we observe a significant downregulation of E-cadherin protein expression in floating SW620 and HeLaF11 cells, whereas C33a cells are E-cadherin negative. There is upregulation of several proteins associated with metastasis, including VEGF. Notably, a significant upregulation of MMP14 (Matrix Metallopeptidase 14) is seen in the floating population of SW620 and C33a cells. In that respect, MMP14 has been shown to decrease cell adhesion and provide anoikis resistance in SW620 cells [35].

Several different signaling pathways were shown to be significantly different between floating and adherent cells. Our trials of inhibiting mTOR and ROCK signaling showed the floating cells were more sensitive to these drugs. Clearly, additional work is needed to understand the underlying molecular mechanism behind this. However, it is encouraging and suggests this model can be used as a very fast preliminary screening method to test how specific inhibitors affect the two cell populations differently, which may give insight into their putative promise in cancer and metastasis in particular. Inhibitors of mTOR, for example, are currently being evaluated in the clinic, and the observation that floating cells are particularly sensitive is of interest, as we suggest it can be a potent inhibitor of metastasis if administered during the time window of cell detachment in a tumor. Both in vivo and in vitro studies also implicate ROCK as a potential target for cancer treatment, especially to prevent metastasis. The fact that both pathways are significantly involved in metastasis and their chemical inhibition affects floating cells specifically suggest that the “floating cell model” studied here may have the potential for fast and cheaper preliminary screenings of anti-metastatic drugs. Our observation that mTOR and ROCK inhibitors are more effective in the floating subpopulation of cells is supported by the fact that cell detachment has been shown to prime SW620 cells to anoikis [36]. However, additional work will determine the robustness of this model.

Changes in pathways related to apoptosis were also observed. We analyzed the percentage of cells undergoing Caspase-3/7 apoptosis and apoptosis with the externalization of phosphatidylserine. In both cases, the percentage was lower than 4% and with no differences between floating and adherent cells. These results suggest very low levels of apoptosis under normal cell culture conditions. In the case of the floating cells, these results suggest that they are resistant to anoikis because they do not present programmed cell death when they detach. Indeed, SW620 cells derived from a metastatic site are resistant to anoikis, whereas the SW420 cell line derived from the primary tumor was sensitive [36]. However, ROCK and mTOR inhibition significantly induced both caspase-dependent and caspase-independent apoptosis specifically in the floating cell population. Further screening of drugs may identify links between cell detachment and sensitivity to certain drugs. Another observation was the enhanced clustering of floating cells culturing in “suspension culture dishes” that prevents adherence to the dish. Whether major gene-expression changes occur under these conditions, and whether this further affects drug sensitivity and anoikis, would be an interesting future question to address.

In the tumor microenvironment, alteration in the expression of many pro and antimetastatic genes takes place at the molecular level, and this is often due to cellular stress that further aggravates the metastatic cascade and advancement of the disease. Hypoxia is one of these stress factors that promotes metastatic progression [27]. Again, this condition increased the ratio of floating cells, further consolidating the validity of this model. Mechanistically, hypoxia has been shown to influence the invasive and migratory behavior of cancer cells via EMT (trans-differentiation of cells in order to acquire plastic and mobile abilities), a process that alters their gene expression prior to migration. Indeed, we see downregulation of the EMT marker E-cadherin in floating cells, which may in part be due to downregulated NM23-H1 expression, a feature we also observed in hypoxia [29,30]. Using the RNAseq data, there is now an opportunity to screen some of the other hypoxia-associated genes that are differentially expressed in floating cells to assay their causative implication in the process of detachment.

This work has demonstrated that many of the characteristics of the floating cells resemble critical features of metastatic cells, such as detachment or anoikis resistance. It would also be of interest to compare the gene expression of these floating cells with that of typical circulating tumor cells, such as leukemia cells. However, obviously, a model of metastasis based on these cells has some limitations. On the one hand, it is a highly simplistic in vitro 2-D model that does not accurately resemble the particularities of a tumor microenvironment such as native morphology and physiological processes of tumor cells, features that more complex 3-D models are trying to address [4]. While in vitro models offer clear advantages over in vivo models of metastasis, most notably cost, reproducibility and high throughput applications, they remain surrogate assays of metastatic function that merely provide a rapid, simplified system that can be manipulated to investigate factors regulating specific steps of the metastatic cascade or test the efficacy of inhibitors. Yet, these features are critical in the early steps of evaluating drugs and pathways. The sheer simplicity of this system allows any lab to perform similar experiments or validate any gene or pathway of interest such as those disclosed here from the RNAseq analysis. It would be necessary to validate the results in other models in vitro and in vivo that fulfil some of the characteristics that this model of floating cells lacks, such as cooperation with other cells and tissues, and subsequently also in clinical specimens. We believe however that this model, and the associated RNAseq data, in combination with other more complex models and systems, will be useful in the critical search for novel anti-metastatic targets.

## 4. Materials and Methods

### 4.1. Cell Culture

C33a (ATCC^®^ HTB-31) cervical carcinoma cells, SW620 (ATCC^®^ CCL-227) colorectal adenocarcinoma cells and HeLa (ATCC^®^ CCL-2) cervical adenocarcinoma cells were obtained from ATCC (LGC Standards, Barcelona, Spain) and cultured as recommended using standard procedures. Briefly, cells were cultured in Dulbecco’s modified Eagle’s minimum essential medium (DMEM, Thermo Fisher Scientific, Waltham, MA, USA) supplemented with 10% fetal bovine serum (FBS, Labclinics, Barcelona, Spain) and 1% penicillin/streptomycin (P/S, Thermo Fisher Scientific, Waltham, MA, USA). Cells were cultured in a 37 ºC humidified incubator in an atmosphere of 5% CO2. TrypLE Express (Thermo Fisher Scientific, Waltham, MA, USA) was added to detach cells from the plate.

### 4.2. RNA Isolation and Sequencing

The floating cell population and the adherents cell population from three 10 cm dishes were isolated and pooled for each cell line. RNA was isolated using an RNAeasy Plus Mini Kit (Qiagen, (Werfen, Barcelona, Spain) Cat No: 74134) that specifically removes any genomic DNA contamination using gEliminator Spin-columns. The quantity was analyzed by Nanodrop ND-1000 Analyzer and the quality was analyzed with a Bioanalyzer 2100 (Agilent Technologies, Santa Clara, CA, USA). Minimum 5 ug of RNA, with a minimum RIN (RNA integrity number) of 7, was shipped to Beijing Genomics Institute (BGI), Shenzhen, China, using RNA stable tubes (Biomatrica (San Diego, CA, USA) Cat. No 93220-001). BGI Beijing performed further internal quality control assays. Gene expression profiling by RNA sequencing was performed entirely by BGI as previously described [37,38]. Briefly: After extracting the total RNA from the samples, mRNA is enriched by using the oligo(dT) magnetic beads. Adding the fragmentation buffer, the mRNA is interrupted to short fragments (about 200 bp), then the first strand cDNA is synthesized by random hexamer-primer using the mRNA fragments as templates. Buffer, dNTPs, RNase H and DNA polymerase I are added to synthesize the second strand. The double-strand cDNA is purified with QiaQuick PCR extraction kit (Werfen, Barcelona, Spain) and washed with EB buffer for end repair and single nucleotide A (adenine) addition. Finally, sequencing adaptors are ligated to the fragments. The required fragments are purified by agarose gel electrophoresis and enriched by PCR amplification. The library products are ready for sequencing analysis via Illumina HiSeq™ 2000. Clean reads were mapped to reference sequences using SOAPaligner/soap2 [39]. Mismatches no more than 2 bases were allowed in the alignment. The gene expression level is calculated by using the RPKM [40] method (Reads Per kb per Million reads).

### 4.3. Timelapse Imaging

IncuCyte ZOOM Live-Cell Imaging system (Essen Bioscience, Hertfordshire, UK) was used to obtain images of adherent cells detaching from the plate and floating cells reattaching to the plate. Cells were seeded in a 24-well plate with increasing numbers of cells, from 10,000 per well to 50,000 per well. Then, 24 h later, floating cells were collected from the media and put in another well. The 24-well plate was put in the IncuCyte inside the tissue culture incubator and phase-contrast images were taken every 5 min for 48 h.

### 4.4. Drug Treatments

A total of 500,000 cells/well were seeded in 6-well plates 48 h before the treatment. Drug treatment was applied 6 h before the viability assay was performed. The mTOR pathway inhibitor PP242 (Tocris, Bristol, UK) was used at 100 nM (1:4000), and the ROCK inhibitor Y-27632 (Tocris, Bristol, UK) was used at 20 mM (1:2000). DMSO diluent was used as a control.

### 4.5. Viability Assays

Both cell viability and cell death were independently quantified after drug treatment by means of fluorescent staining, using the ReadyProbes Cell Viability Imaging Kit (Thermo Fisher Scientific, Waltham, MA, USA). After an overnight or 6 h treatment in 6-well plates, floating cells were collected and adherent cells were trypsinized and resuspended in PBS-BSA. One drop of propidium iodide stain (staining in red the nuclei of dead cells with compromised plasma membrane) was added to each 0.5 mL of cells with PBS-BSA. After 15 min incubation, samples were put in a 96-well (100 ul containing 50,000 cells per well) and observed using an Olympus FSX100 fluorescent microscope (Olympus Iberia, Barcelona, Spain) The percentage of dead cells was determined by counting the number of red cells in an area with 100 total cells (seen with phase contrast).

In addition to dead cells, apoptotic cells were determined. Caspase-3 is an early indicator of apoptosis, so CellEvent Caspase-3/7 Green Ready Probe (Thermo Fisher Scientific, Waltham, MA, USA) was used to detect apoptotic cells. CellEvent Caspase-3/7 is a 4-amino acid peptide (DEVD) conjugated with a stain that binds to DNA (this reagent is only fluorescent when bound to DNA). Two drops per mL of PBS-BSA were used and after 15 min of incubation at room temperature cells were viewed at the fluorescence microscope (FSX100 Olympus, (Olympus Iberia, Barcelona, Spain)). The percentage of dead cells due to Caspase-3/7 apoptosis was also determined by counting the number of green cells in an area with 100 total cells (seen with phase contrast). The Thermo Scientific Appliskan (Thermo Fisher Scientific, Waltham, MA, USA) was also used to determine the ratio between dead and total cells. To determine the number of dead cells, a first read was done to a 96-well plate with 100 μL of PBS-BSA with 50,000 per well with 10 ul of propidium iodide (1% diluted in PBS-BSA). Then, 10 ul of TritonX was added to each well, and a second read was done to determine the number of total cells.

### 4.6. Western Blotting and Antibodies

Cells were lysed in RIPA buffer (Santa Cruz Biotechnology, Heidelberg, Germany) and Western blotting was performed as previously described [41]. Protein was quantified using the bicinchoninic acid (BCA) assay kit (Thermo Fisher Scientific, Waltham, MA, USA) and verified by Western blot using anti-actin and anti-vinculin antibodies. The following antibodies and dilutions were used: anti-NM23 (Abcam, Cambridge, UK, 1:700), anti-vinculin (Sigma-Prestige Antibodies, Merck Life Science, Madrid, Spain. 1:200), anti-β-actin (Calbiochem, (Merck Life Science, Madrid, Spain), 1:500), secondary goat anti-rabbit and goat anti-mouse HRP (Thermo Fisher Scientific, Waltham, MA, USA, 1:15,000). Quantification was performed using Image J.

### 4.7. NM23-H1 Knockdown and Overexpression

Cells in 6-well plates were transfected with 2 µg RNA using RNAiMax (Thermo Fisher Scientific, Waltham, MA, USA) following the manufacturer’s recommended standard procedure. The medium was changed 24 h after transfection and 72 h after transfection the amounts of floating and adherent cells were quantified by counting the cells in a Neubauer chamber and a Western blot was performed to check the efficiency of the knock-down. The floating cells obtained after transfection with siRNA control and siRNA NM23 were put in a new 6-well plate, and after 72 h the colony formation capacity was quantified using crystal violet. Then, 1 mL of crystal violet 5% (Sigma-Aldrich, Taufkirchen, Germany) was added to each well, and after 30 min, the cells were washed with PBS and then the crystal violet was quantified adding 100 μL of acetic acid. Then, 200 μL was loaded to a 96 well-plate and read at an absorbance of 590 nm using Epoch Biotek Spectrophotometer (Friedrichshall, Germany).

The retroviral plasmid for overexpression of NM23-H1 (pBABE-hygro NM23 H1 WT) was a gift from Ronald Kahn (Addgene plasmid # 11364; available on http://n2t.net/addgene:11364 last accessed on 2 May 2021; RRID: Addgene_11364) and was generated as described [42]. Retrovirus was produced and used to infect HeLa and C33a cells as previously described [43].

### 4.8. Hypoxia Treatment

A total of 200,000 cells were seeded per well in a 6-well plate and after 48 h were put in an Invivo 200 hypoxia hood (Baker Ruskinn) with an oxygen concentration of 0.5% and a CO_2_ concentration of 5%. After 48 h protein extraction and cell counting was performed.

### 4.9. Flow Cytometry Analysis

106 C33a cells were seeded in 10 cm plates. After 4 days cells were treated with diluted DMSO (as a control), PP242 100 nM or Staurosporine 5 mM (as an apoptotic inducer) overnight. After this period, floating and adherent cells were collected, washed in cold PBS and treated with Annexin V-FITC Apoptosis Detection Kit (Thermo Fisher Scientific, Waltham, MA, USA). The cells were re-centrifuged and after discarding the supernatant, resuspended in 100 μL 1x Binding Buffer; 5 ul of Annexin V-FITC and 5 ul PI Staining Solution were added to each 100 ul of cell suspension. After a 15 min incubation at room temperature, 400 ul of 1x Binding Buffer was added. Finally, the stained cells were analyzed by BD LSRFortessa flow cytometry analyzer (Thermo Fisher Scientific, Waltham, MA, USA), measuring the fluorescence emission at 530 nm and >575 nm.

### 4.10. Statistical Analysis

Values are expressed as means. Statistical analyses and drawing of graphs were done using GraphPad Prism 6.0 (San Diego, CA, USA). Student’s *t*-test with Spearman correlation analysis or one way ANOVA with Tukey’s correction was performed as specified in the figure legends. A *p*-value < 0.05 was considered statistically significant and n.s. was reported for non-statistically significant results (*p* > 0.05). Unless specified, all the experiments were performed at least three independent times.

## 5. Conclusions

Gene expression analysis using RNA seq identified a unique subset of genes differentially expressed between floating and adherent cells isolated from the same tissue culture dish. Comparing three different cell lines, we observed that 122 of these genes were shared. Many of these genes have been associated with the metastatic process. One of these genes, NM23-H1, was tested functionally and was shown to positively regulate the process of detachment and anchorage-independent growth. Pathway analysis also revealed a number of alternations and blocking the mTOR and ROCK pathways resulted in increased cell death, specifically in the cells in suspension, indicative of a therapeutic window. We believe that the coexistence of adherent and floating cells offers a simple but valuable tool for both screening and testing of genes and compounds related to malignancy and, in particular, metastasis.

## Data Availability

All gene expression data available are freely provided in the supplementary Tables. Raw FASTQ-data for databases are not available as these were not provided by the sequencing company.

## Supplementary Materials

The following are available online at https://www.mdpi.com/article/10.3390/ijms22094929/s1, Figure S1: Cell viability and cell proliferation of adherent versus floating cells, Figure S2: Still photos derived from supplementary videos 1–2, Figure S3: Hallmarks of cancer pathways derived from gene pattern analysis of RNAseq data, Figure S4: Annotation clusters derived from RNAseq analysis using DAVID pathway analysis, Figure S5: GO Pathway analysis of RNAseq data, Figure S6: Flow cytometry analysis of cell death and apoptosis, Table S1: Gene expression of all genes in all cell lines, Table S2: Differentially expressed genes comparing adherent and floating C33a cells, Table S3: Differentially expressed genes comparing adherent and floating SW620 cells, Table S4: Differentially expressed genes comparing adherent and floating HeLaF11 cells, Table S5: Shared differentially expressed genes from adherent and floating C33a/SW620/HeLaF11 cells, Table S6: Differentially expressed genes comparing adherent HeLa cells and adherent HeLaF11 cells, Video S1: Time-lapse imaging of C33a cells, Video S2: Time-lapse imaging of SW620 cells.

## References

[1] Buchheit C.L., Weigel K.J., Schafer Z.T. (2014). Cancer Cell Survival during Detachment from the ECM: Multiple Barriers to Tumour Progression. Nat. Rev. Cancer.

[2] Bersini S., Jeon J.S., Moretti M., Kamm R.D. (2014). In Vitro Models of the Metastatic Cascade: From Local Invasion to Extravasation. Drug Discov. Today.

[3] Weiswald L.-B., Bellet D., Dangles-Marie V. (2015). Spherical Cancer Models in Tumor Biology. Neoplasia.

[4] Albritton J.L., Miller J.S. (2017). 3D Bioprinting: Improving in Vitro Models of Metastasis with Heterogeneous Tumor Microenvironments. Dis. Model. Mech..

[5] Perkhofer L., Frappart P.-O., Müller M., Kleger A. (2018). Importance of Organoids for Personalized Medicine. Per. Med..

[6] Strong A.D., Daniels R.L. (2017). Live-Cell Calcium Imaging of Adherent and Non-Adherent GL261 Cells Reveals Phenotype-Dependent Differences in Drug Responses. BMC Cancer.

[7] Wheeler L.J., Watson Z.L., Qamar L., Yamamoto T.M., Sawyer B.T., Sullivan K.D., Khanal S., Joshi M., Ferchaud-Roucher V., Smith H. (2019). Multi-Omic Approaches Identify Metabolic and Autophagy Regulators Important in Ovarian Cancer Dissemination. iScience.

[8] Prud’homme G.J. (2012). Cancer Stem Cells and Novel Targets for Antitumor Strategies. Curr. Pharm. Des..

[9] Chen H.-C., Chou A.S.-B., Liu Y.-C., Hsieh C.-H., Kang C.-C., Pang S.-T., Yeh C.-T., Liu H.-P., Liao S.-K. (2011). Induction of Metastatic Cancer Stem Cells from the NK/LAK-Resistant Floating, but Not Adherent, Subset of the UP-LN1 Carcinoma Cell Line by IFN-γ. Lab. Investig..

[10] Zheng Q., Gong F., Xu Y., Zheng T., Ying M. (2011). Floating Cells with Stem Cell Properties in Gastric Cell Line SGC-7901. Tumori.

[11] He Q.-Z., Luo X.-Z., Wang K., Zhou Q., Ao H., Yang Y., Li S.-X., Li Y., Zhu H.-T., Duan T. (2014). Isolation and Characterization of Cancer Stem Cells from High-Grade Serous Ovarian Carcinomas. Cell. Physiol. Biochem..

[12] Shi C.-J., Gao J., Wang M., Wang X., Tian R., Zhu F., Shen M., Qin R.-Y. (2011). CD133(+) Gallbladder Carcinoma Cells Exhibit Self-Renewal Ability and Tumorigenicity. World J. Gastroenterol..

[13] Sagawa Y., Fujitoh A., Nishi H., Ito H., Yudate T., Isaka K. (2011). Establishment of Three Cisplatin-Resistant Endometrial Cancer Cell Lines Using Two Methods of Cisplatin Exposure. Tumor Biol..

[14] Doyle L.A., Borges M., Hussain A., Elias A., Tomiyasu T. (1990). An Adherent Subline of a Unique Small-Cell Lung Cancer Cell Line Downregulates Antigens of the Neural Cell Adhesion Molecule. J. Clin. Invest..

[15] Kraus A.C., Ferber I., Bachmann S.-O., Specht H., Wimmel A., Gross M.W., Schlegel J., Suske G., Schuermann M. (2002). In Vitro Chemo- and Radio-Resistance in Small Cell Lung Cancer Correlates with Cell Adhesion and Constitutive Activation of AKT and MAP Kinase Pathways. Oncogene.

[16] Gomez-Casal R., Bhattacharya C., Ganesh N., Bailey L., Basse P., Gibson M., Epperly M., Levina V. (2013). Non-Small Cell Lung Cancer Cells Survived Ionizing Radiation Treatment Display Cancer Stem Cell and Epithelial-Mesenchymal Transition Phenotypes. Mol. Cancer.

[17] Krohn A., Ahrens T., Yalcin A., Plönes T., Wehrle J., Taromi S., Wollner S., Follo M., Brabletz T., Mani S.A. (2014). Tumor Cell Heterogeneity in Small Cell Lung Cancer (SCLC): Phenotypical and Functional Differences Associated with Epithelial-Mesenchymal Transition (EMT) and DNA Methylation Changes. PLoS ONE.

[18] Kawaguchi T. (2005). Cancer Metastasis: Characterization and Identification of the Behavior of Metastatic Tumor Cells and the Cell Adhesion Molecules, Including Carbohydrates. Curr. Drug Targets Cardiovasc. Haematol. Disord..

[19] Thorne H.J., Jose D.G., Zhang H.Y., Dempsey P.J., Whitehead R.H. (1987). Epidermal Growth Factor Stimulates the Synthesis of Cell-Attachment Proteins in the Human Breast Cancer Cell Line PMC42. Int. J. Cancer.

[20] Jensen K., Patel A., Klubo-Gwiezdzinska J., Bauer A., Vasko V. (2011). Inhibition of Gap Junction Transfer Sensitizes Thyroid Cancer Cells to Anoikis. Endocr. Relat. Cancer.

[21] Nagaprashantha L., Vartak N., Awasthi S., Awasthi S., Singhal S.S. (2012). Novel Anti-Cancer Compounds for Developing Combinatorial Therapies to Target Anoikis-Resistant Tumors. Pharm. Res..

[22] Liu L., Li M., Zhang C., Zhang J., Li G., Zhang Z., He X., Fan M. (2018). Prognostic Value and Clinicopathologic Significance of nm23 in Various Cancers: A Systematic Review and Meta-Analysis. Int. J. Surg..

[23] Marino N., Nakayama J., Collins J.W., Steeg P.S. (2012). Insights into the Biology and Prevention of Tumor Metastasis Provided by the Nm23 Metastasis Suppressor Gene. Cancer Metastasis Rev..

[24] Boissan M., Schlattner U., Lacombe M.-L. (2018). The NDPK/NME Superfamily: State of the Art. Lab. Investig..

[25] Boissan M., De Wever O., Lizarraga F., Wendum D., Poincloux R., Chignard N., Desbois-Mouthon C., Dufour S., Nawrocki-Raby B., Birembaut P. (2010). Implication of Metastasis Suppressor NM23-H1 in Maintaining Adherens Junctions and Limiting the Invasive Potential of Human Cancer Cells. Cancer Res..

[26] Bago R., Pavelić J., Maravić Vlahovicek G., Bosnar M.H. (2009). Nm23-H1 Promotes Adhesion of CAL 27 Cells in Vitro. Mol. Carcinog..

[27] Gilkes D.M., Semenza G.L., Wirtz D. (2014). Hypoxia and the Extracellular Matrix: Drivers of Tumour Metastasis. Nat. Rev. Cancer.

[28] Reich M., Liefeld T., Gould J., Lerner J., Tamayo P., Mesirov J.P. (2006). GenePattern 2.0. Nat. Genet..

[29] Rasool R.U., Nayak D., Chakraborty S., Jamwal V.L., Mahajan V., Katoch A., Faheem M.M., Iqra Z., Amin H., Gandhi S.G. (2017). Differential Regulation of NM23-H1 under Hypoxic and Serum Starvation Conditions in Metastatic Cancer Cells and Its Implication in EMT. Eur. J. Cell Biol..

[30] Wu C.-E., Zhuang Y.-W., Zhou J.-Y., Liu S.-L., Zou X., Wu J., Wang R.-P., Shu P. (2019). Nm23-H1 Inhibits Hypoxia Induced Epithelial-Mesenchymal Transition and Stemness in Non-Small Cell Lung Cancer Cells. Biol. Chem..

[31] Yao X., Jennings S., Ireland S.K., Pham T., Temple B., Davis M., Chen R., Davenport I., Biliran H. (2014). The Anoikis Effector Bit1 Displays Tumor Suppressive Function in Lung Cancer Cells. PLoS ONE.

[32] Paolo P., Elisa G., Paola C. (2013). Anoikis Molecular Pathways and Its Role in Cancer Progression. Biochim. Biophys. Acta.

[33] Ng T.L., Leprivier G., Robertson M.D., Chow C., Martin M.J., Laderoute K.R., Davicioni E., Triche T.J., Sorensen P.H.B. (2012). The AMPK Stress Response Pathway Mediates Anoikis Resistance through Inhibition of mTOR and Suppression of Protein Synthesis. Cell Death Differ..

[34] Donat U., Rother J., Schäfer S., Hess M., Härtl B., Kober C., Langbein-Laugwitz J., Stritzker J., Chen N.G., Aguilar R.J. (2014). Characterization of Metastasis Formation and Virotherapy in the Human C33A Cervical Cancer Model. PLoS ONE.

[35] Liu Y., Zhang Y., Wu H., Li Y., Zhang Y., Liu M., Li X., Tang H. (2017). miR-10a Suppresses Colorectal Cancer Metastasis by Modulating the Epithelial-to-Mesenchymal Transition and Anoikis. Cell Death Dis..

[36] Maamer-Azzabi A., Ndozangue-Touriguine O., Bréard J. (2013). Metastatic SW620 Colon Cancer Cells Are Primed for Death When Detached and Can Be Sensitized to Anoikis by the BH3-Mimetic ABT-737. Cell Death Dis..

[37] Ho D.W.Y., Yang Z.F., Yi K., Lam C.T., Ng M.N.P., Yu W.C., Lau J., Wan T., Wang X., Yan Z. (2012). Gene Expression Profiling of Liver Cancer Stem Cells by RNA-Sequencing. PLoS ONE.

[38] Liu B., Jiang G., Zhang Y., Li J., Li X., Yue J., Chen F. (2011). Analysis of Transcriptome Differences between Resistant and Susceptible Strains of the Citrus Red Mite Panonychus Citri (Acari: Tetranychidae). PLoS ONE.

[39] Li R., Yu C., Li Y., Lam T.-W., Yiu S.-M., Kristiansen K., Wang J. (2009). SOAP2: An Improved Ultrafast Tool for Short Read Alignment. Bioinformatics.

[40] Mortazavi A., Williams B.A., McCue K., Schaeffer L., Wold B. (2008). Mapping and Quantifying Mammalian Transcriptomes by RNA-Seq. Nat. Methods.

[41] Macdonald A.I., Sun P., Hernandez-Lopez H., Aasen T., Hodgins M.B., Edward M., Roberts S., Massimi P., Thomas M., Banks L. (2012). A Functional Interaction between the MAGUK Protein hDlg and the Gap Junction Protein Connexin 43 in Cervical Tumour Cells. Biochem. J..

[42] Zhu J.-H., Tseng Y., Kantor J.D., Rhodes C.J., Zetter B.R., Moyers J.S., Kahn C.R. (1999). Interaction of the Ras-Related Protein Associated with Diabetes Rad and the Putative Tumor Metastasis Suppressor NM23 Provides a Novel Mechanism of GTPase Regulation. Proc. Natl. Acad. Sci. USA.

[43] Aasen T., Izpisúa Belmonte J.C. (2010). Isolation and Cultivation of Human Keratinocytes from Skin or Plucked Hair for the Generation of Induced Pluripotent Stem Cells. Nat. Protoc..

