# Supplementary material for: Spontaneous Cell Detachment and Reattachment in Cancer Cell Lines: An In Vitro Model of Metastasis and Malignancy
Source: Int J Mol Sci. 2021 May 6;22(9):4929. doi: 10.3390/ijms22094929 (PMC8124513; doi:10.3390/ijms22094929)
Supplement: Supplementary file 1 [file ijms-22-04929-s001.zip › Supplementary FiguresS1-6.pdf]

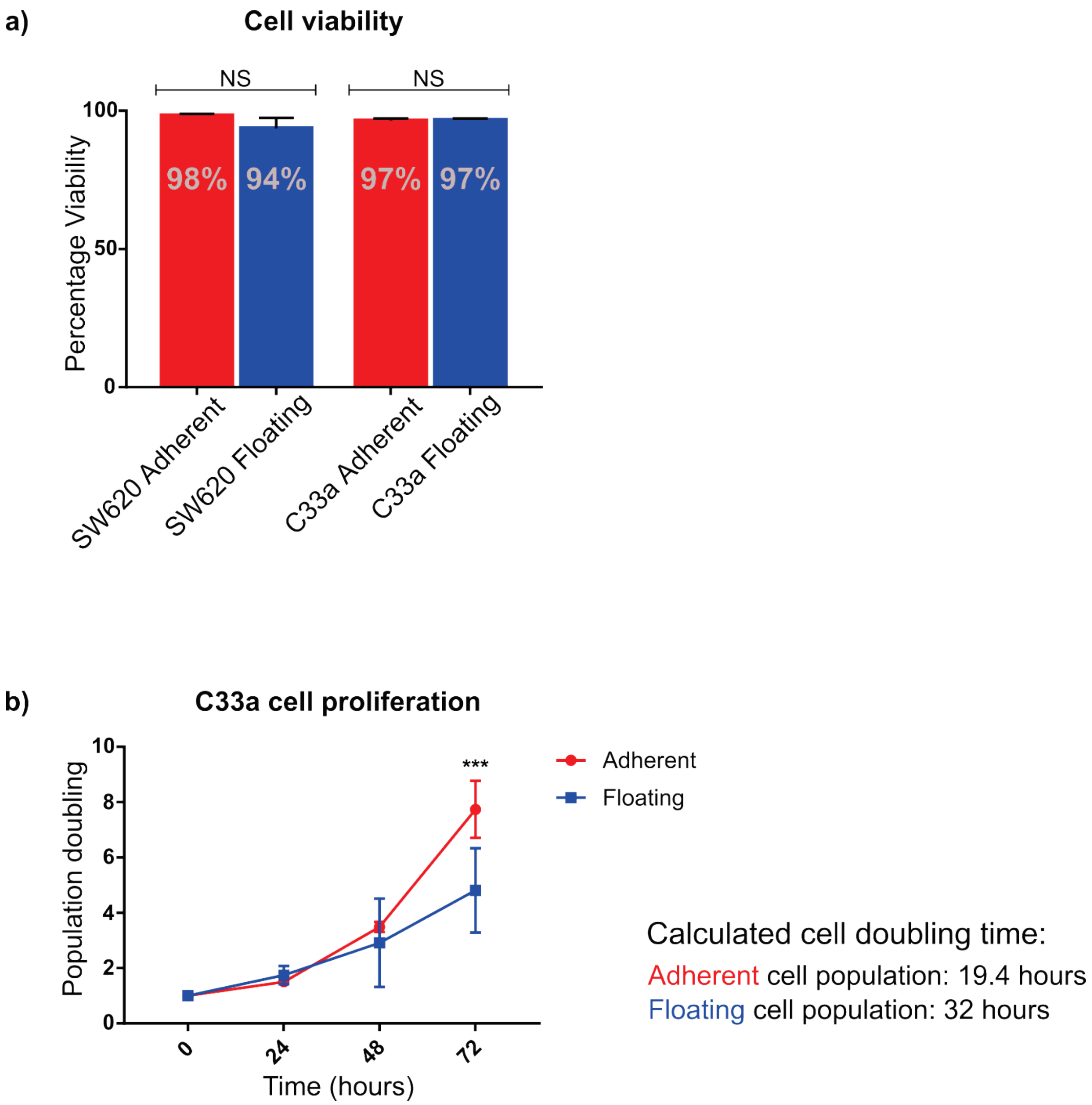


**Supplementary Figure S1. (a)** Cell viability of adherent and floating SW620 and C33a cells under normal conditions. **(b)** Cell proliferation and calculated cell doubling time of C33a cells.

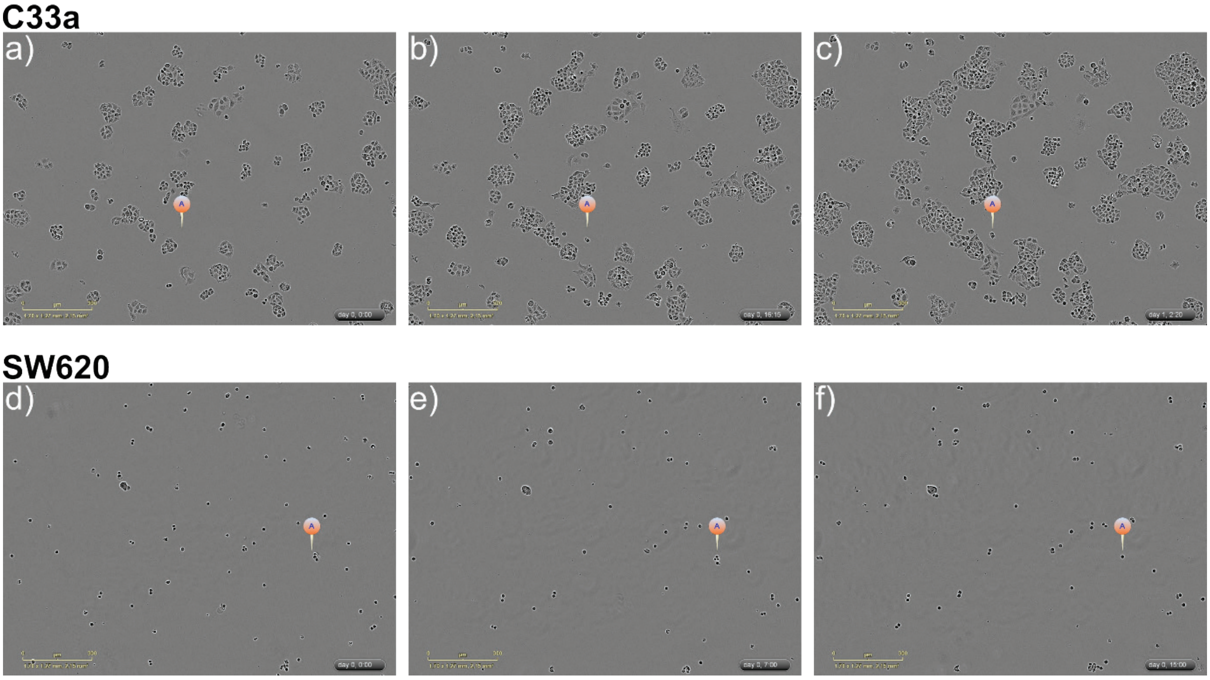


**Supplementary Figure S2.** Still photos derived from Supplementary Videos 1-2. C33a cells imaged at 0 hours **(a)**, 15 hours **(b)** and 26 hours **(c)**, showing attachment and division of a cell. SW620 cells at 0 hours **(d)**, 7 hours **(e)** and 15 hours **(f)**, showing division and detachment of cells.

| Gene Set Name [# Genes (K)] | Description | # Genes in Overlap (k) | k/K | p-value | FDR q-value |
|---|---|---|---|---|---|
| HALLMARK_HYPOXIA [200] | Genes up-regulated in response to low oxygen levels (hypoxia). | 22 | | $2.71\ e^{-21}$ | $1.35\ e^{-19}$ |
| HALLMARK_MYOGENESIS [200] | Genes involved in development of skeletal muscle (myogenesis). | 13 | | $3.39\ e^{-10}$ | $8.48\ e^{-9}$ |
| HALLMARK_GLYCOLYSIS [200] | Genes encoding proteins involved in glycolysis and gluconeogenesis. | 12 | | $4.05\ e^{-9}$ | $5.07\ e^{-8}$ |
| HALLMARK_IL2_STAT5_SIGNALING [200] | Genes up-regulated by STAT5 in response to IL2 stimulation. | 12 | | $4.05\ e^{-9}$ | $5.07\ e^{-8}$ |
| HALLMARK_TNFA_SIGNALING_VIA_NFKB [200] | Genes regulated by NF-kB in response to TNF [GeneID=7124]. | 11 | | $4.44\ e^{-8}$ | $4.44\ e^{-7}$ |
| HALLMARK_APOPTOSIS [161] | Genes mediating programmed cell death (apoptosis) by activation of caspases. | 10 | | $5.87\ e^{-8}$ | $4.89\ e^{-7}$ |
| HALLMARK_P53_PATHWAY [200] | Genes involved in p53 pathways and networks. | 10 | | $4.42\ e^{-7}$ | $3.16\ e^{-6}$ |
| HALLMARK_HEME_METABOLISM [200] | Genes involved in metabolism of heme (a cofactor consisting of iron and porphyrin) and erythroblast differentiation. | 9 | | $3.98\ e^{-6}$ | $2.49\ e^{-5}$ |
| HALLMARK_EPITHELIAL_MESENCHYMAL_TRANSITION [200] | Genes defining epithelial-mesenchymal transition, as in wound healing, fibrosis and metastasis. | 7 | | $2.28\ e^{-4}$ | $9.49\ e^{-4}$ |
| HALLMARK_ESTROGEN_RESPONSE_LATE [200] | Genes defining late response to estrogen. | 7 | | $2.28\ e^{-4}$ | $9.49\ e^{-4}$ |

**Supplementary Figure S3.** Hallmarks of cancer with lowest p-values related with the 267 genes that are differentially expressed between floating and adherent SW620 and C33a cells. Note the highly significant correlation with Hallmarks of Hypoxia (red box).Table obtained at: http://genepattern.broadinstitute.org.

| Annotation Cluster 1 | Enrichment Score: 2.21 | G | | Count | P_Value | Benjamini |
|---|---|---|---|---|---|---|
| KEGG_PATHWAY | PI3K-Akt signaling pathway | RT | | 17 | 2.1E-5 | 3.4E-3 |
| KEGG_PATHWAY | ECM-receptor interaction | RT | | 9 | 2.6E-5 | 2.2E-3 |
| GOTERM_BP_DIRECT | extracellular matrix organization | RT | | 12 | 7.8E-5 | 9.5E-2 |
| GOTERM_BP_DIRECT | cell-matrix adhesion | RT | | 8 | 2.2E-4 | 1.3E-1 |
| GOTERM_CC_DIRECT | integrin complex | RT | | 5 | 4.3E-4 | 1.0E-1 |
| GOTERM_BP_DIRECT | integrin-mediated signaling pathway | RT | | 7 | 2.3E-3 | 5.2E-1 |
| KEGG_PATHWAY | Focal adhesion | RT | | 10 | 2.3E-3 | 9.0E-2 |
| UP_KEYWORDS | Integrin | RT | | 5 | 2.6E-3 | 8.1E-2 |
| KEGG_PATHWAY | Regulation of actin cytoskeleton | RT | | 10 | 2.7E-3 | 8.5E-2 |

**Supplementary Figure S4.** Annotation clusters with lowest p-values related with the 267 genes that are differentially expressed between floating and adherent SW620 and C33a cells. Notably, many of these pathways are centrally regulated by Rho signalling including via ROCK. Table obtained at: http://david.abcc.ncifcrf.gov

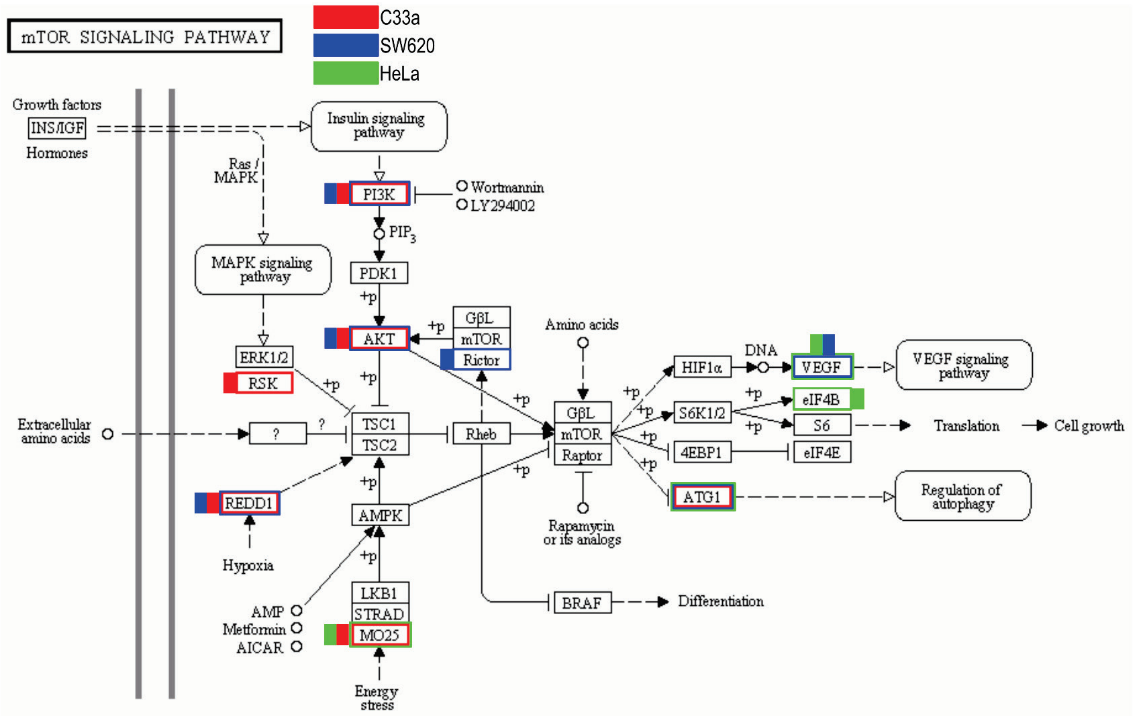


**Supplementary Figure S5.** *GO Pathway analysis.* Although mTOR is not identified as a DEG in this study, a number of DEGs are found in all three cell lines with respect to upstream and downstream genes associated with this pathway

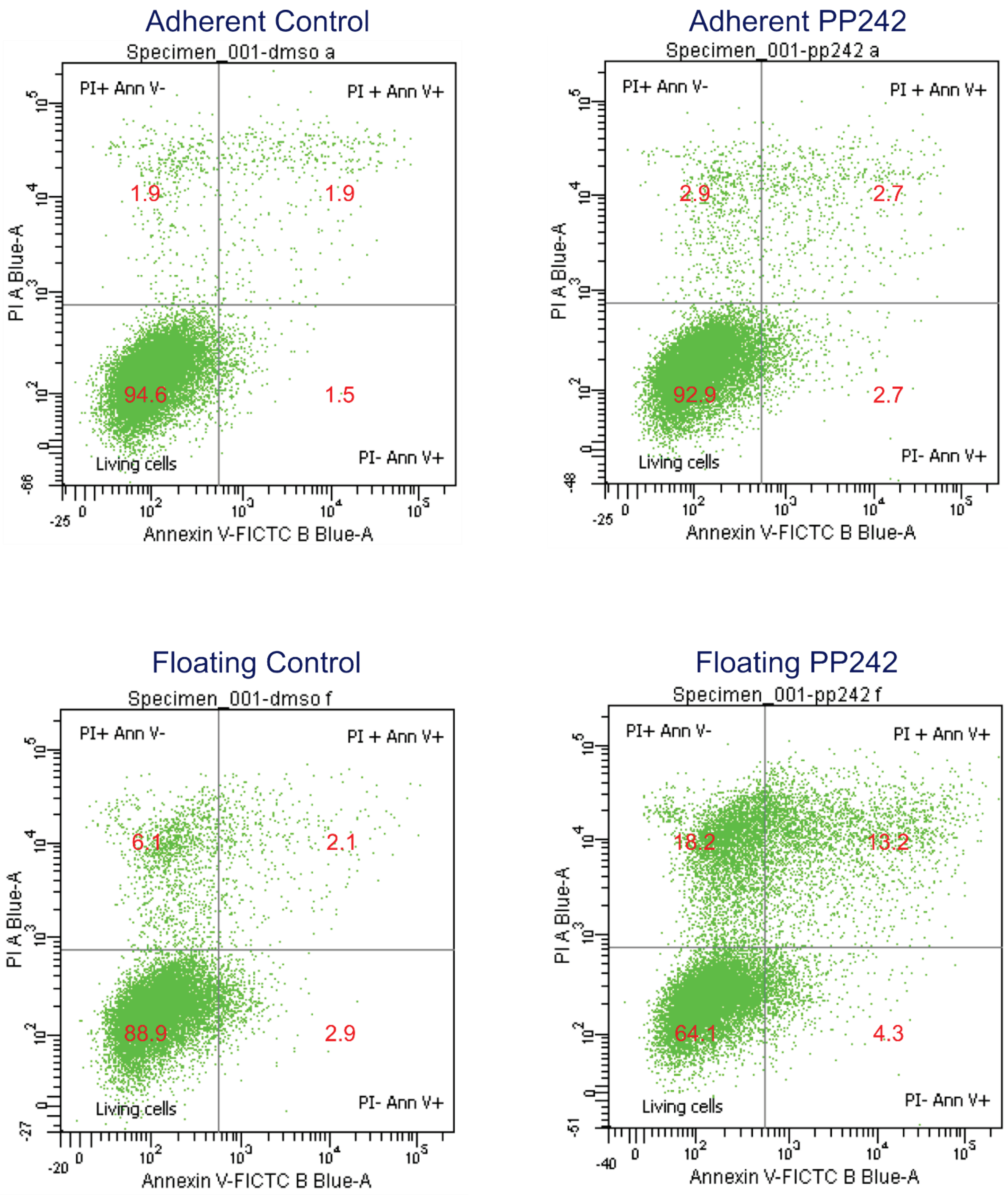


**Supplementary Figure S6.** Flow cytometry analysis of C33a cells using an Annexin V-FITC Apoptosis Detection Kit. **(a)** Adherent cells treated with DMSO. **(b)** Adherent cells treated with PP242. **(c)** Floating cells treated with DMSO. **(d)** Floating cells treated with PP242.
